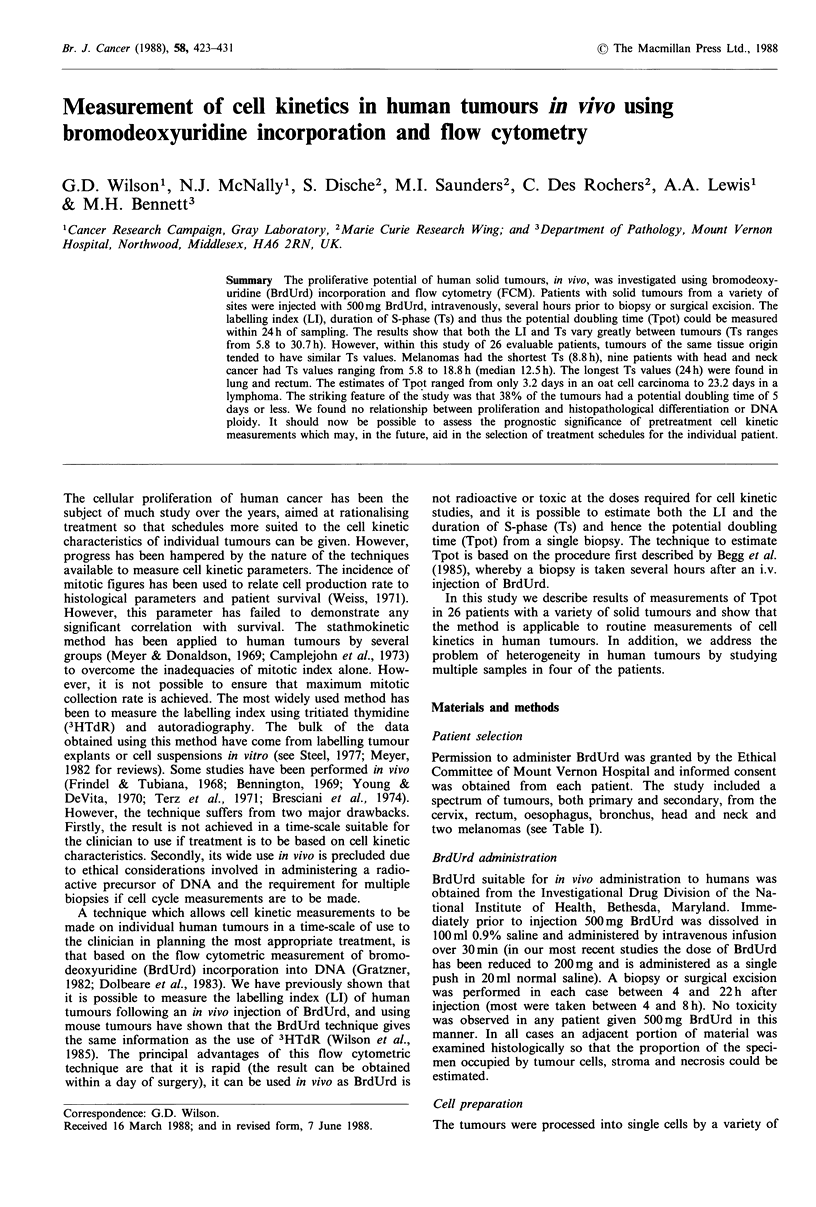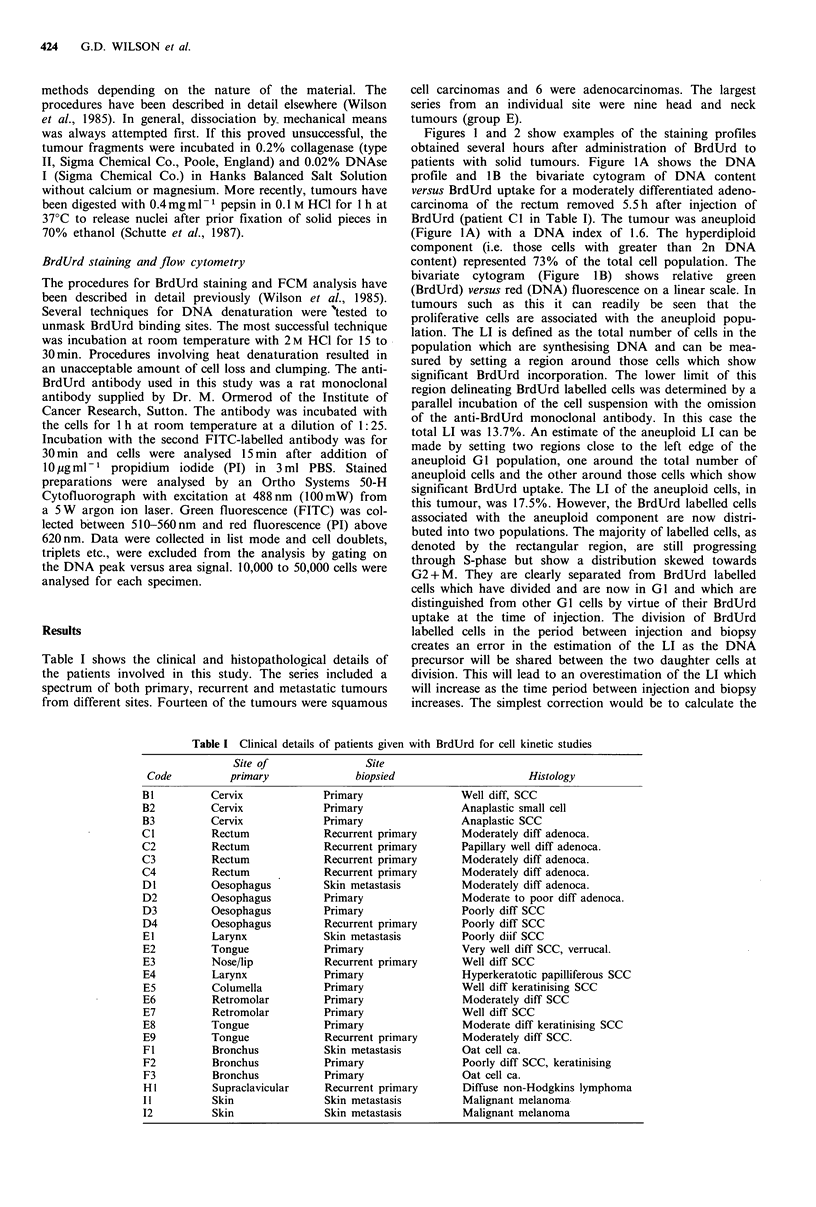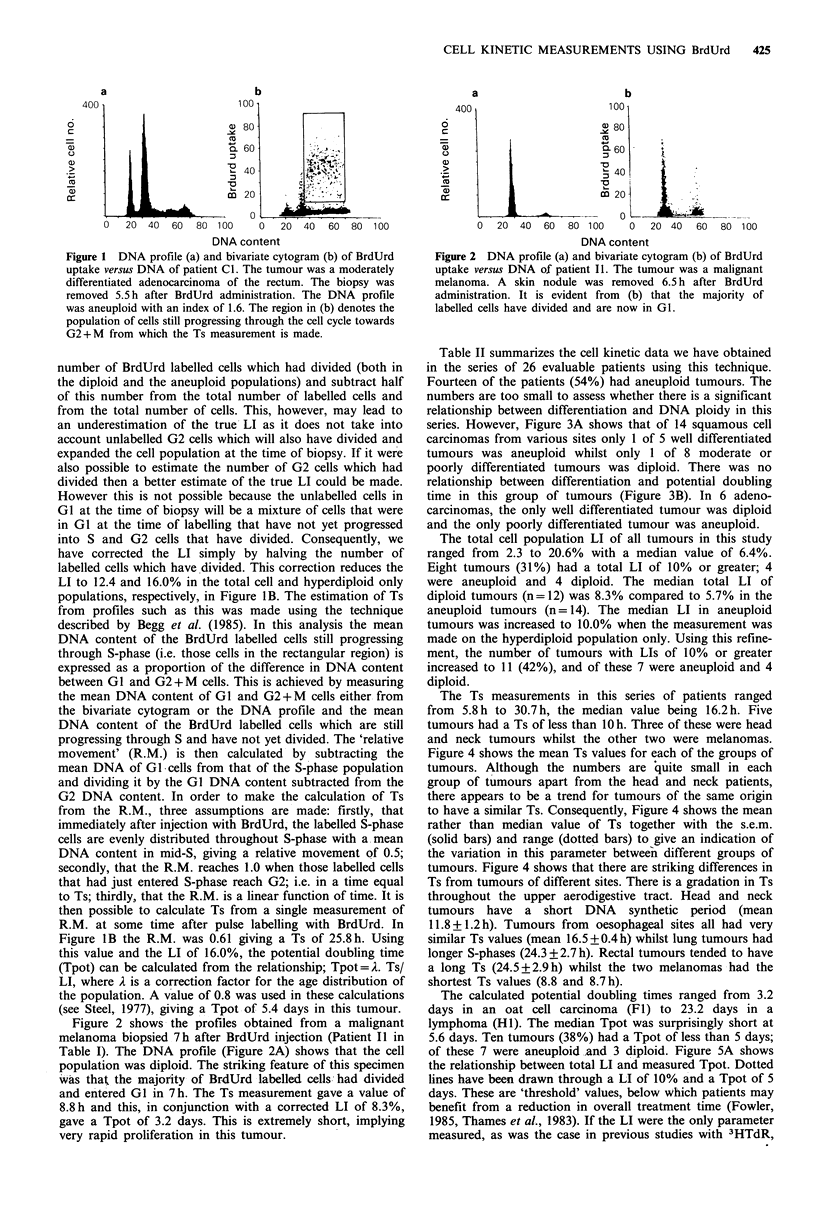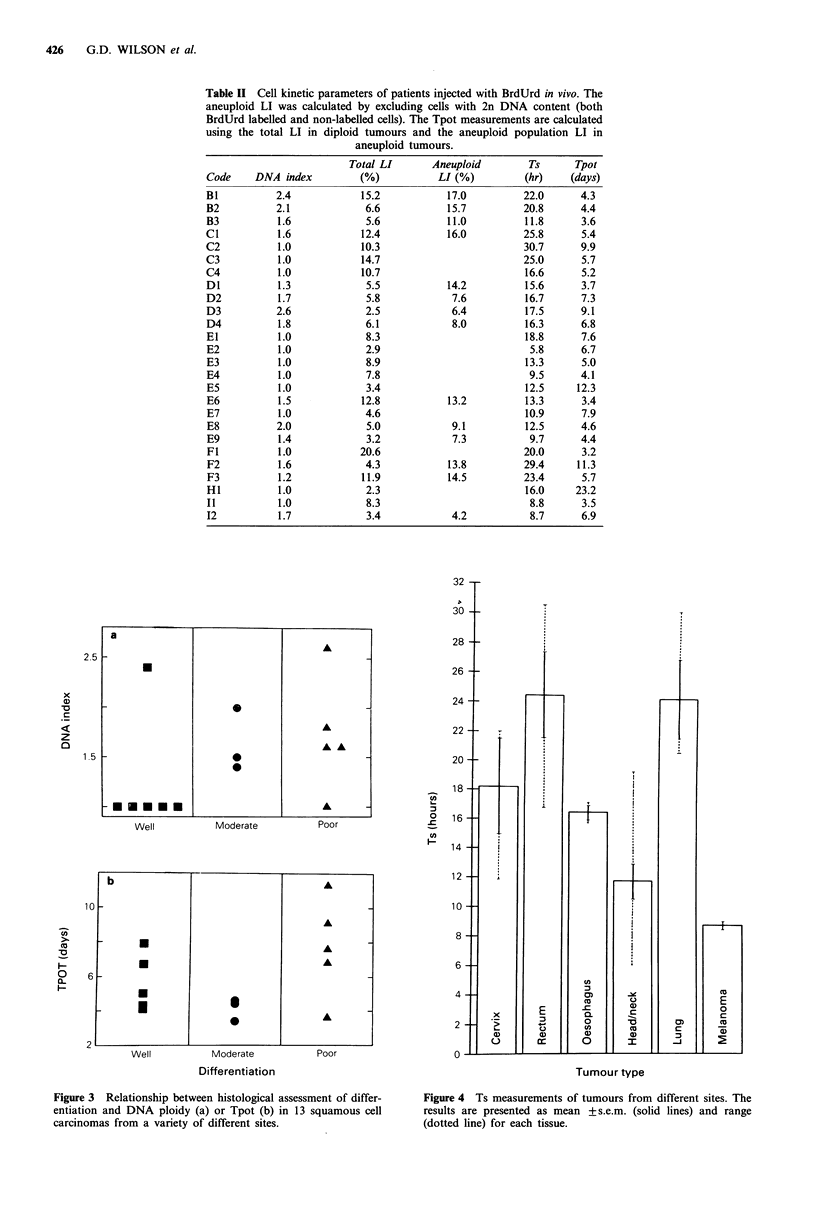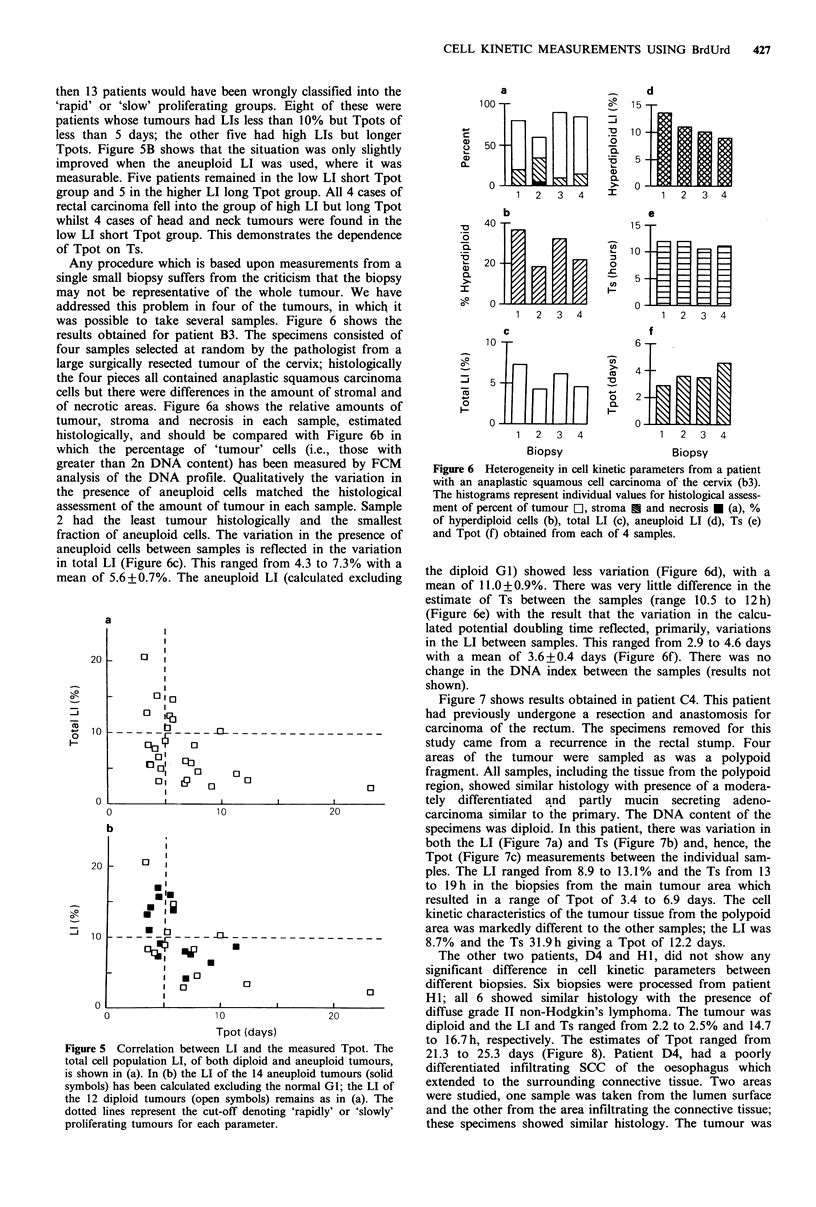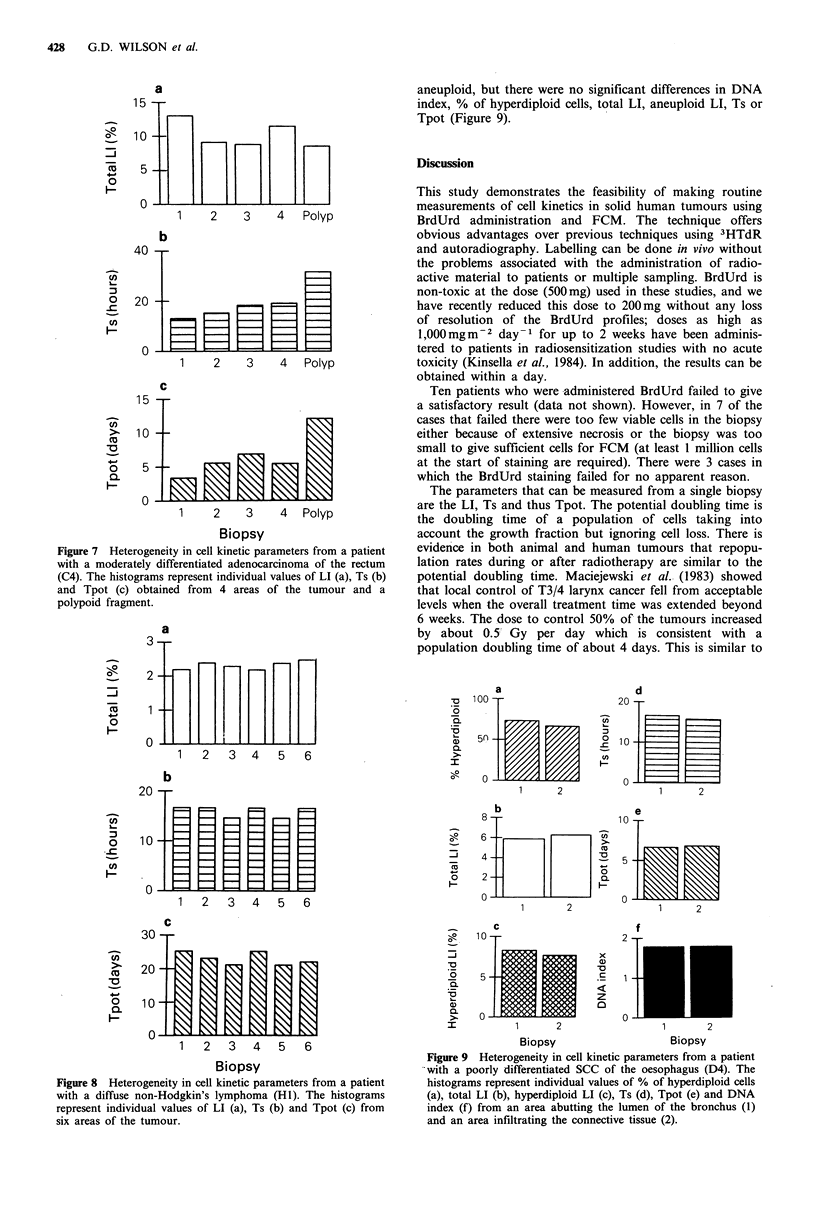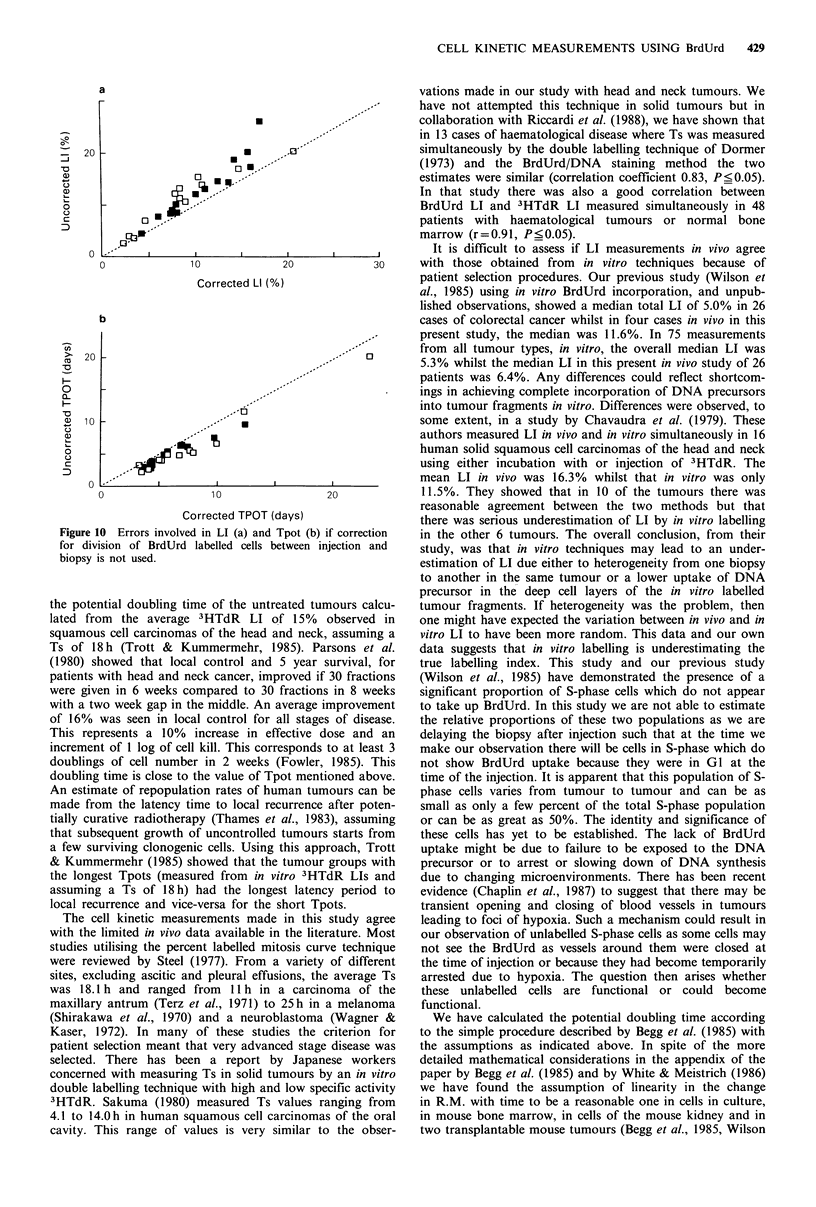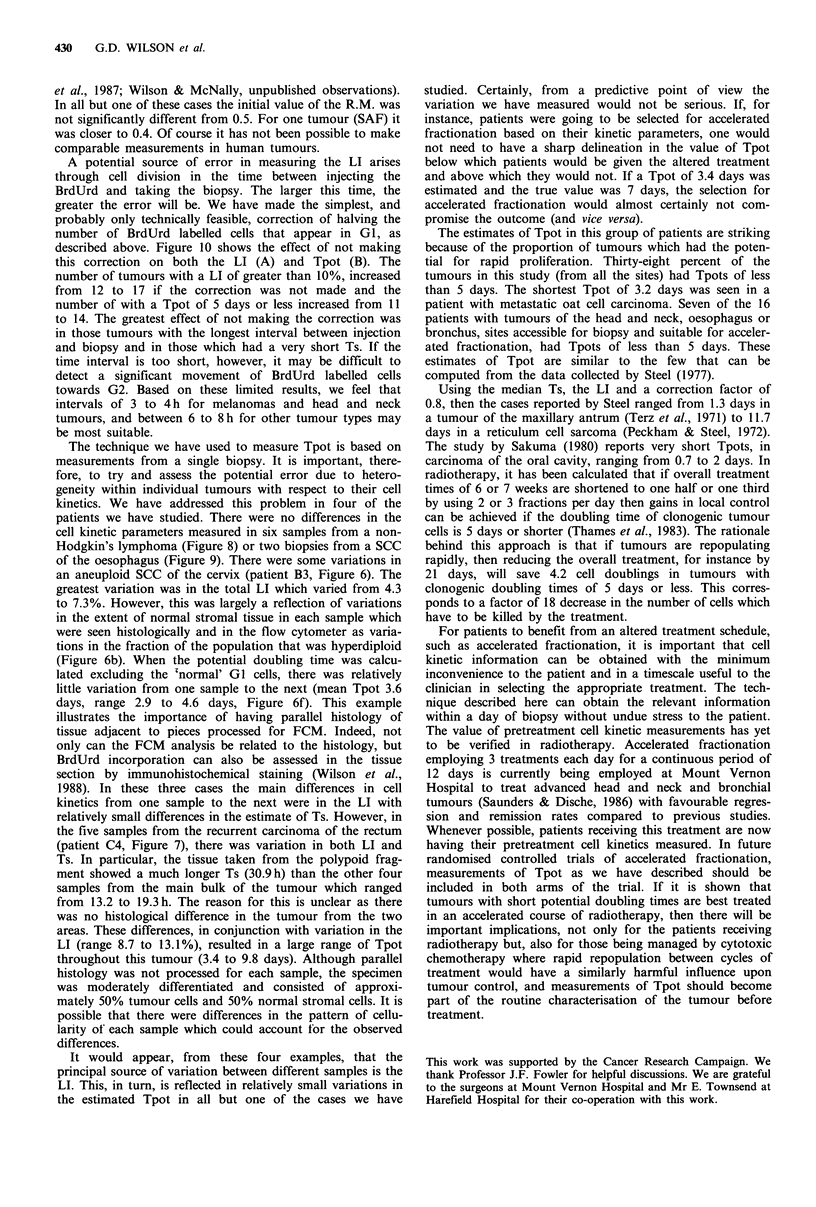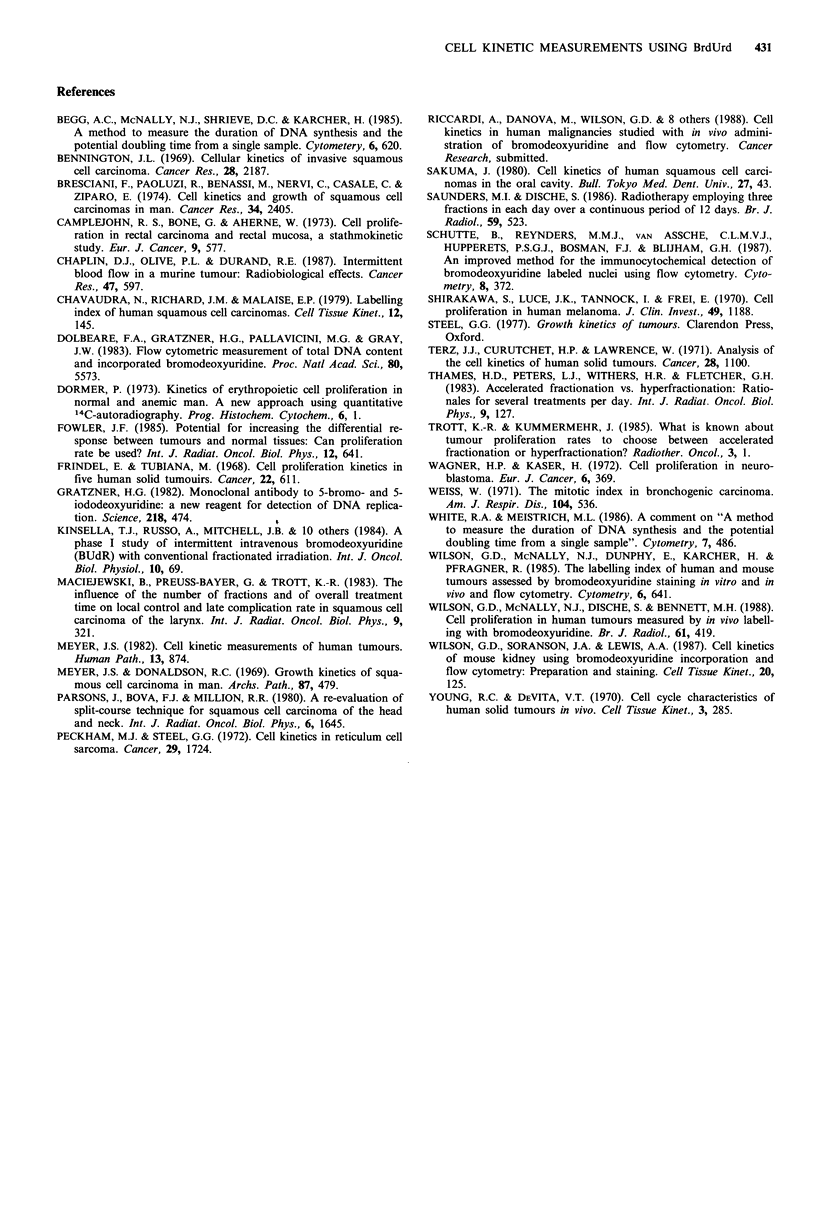# Measurement of cell kinetics in human tumours in vivo using bromodeoxyuridine incorporation and flow cytometry.

**DOI:** 10.1038/bjc.1988.234

**Published:** 1988-10

**Authors:** G. D. Wilson, N. J. McNally, S. Dische, M. I. Saunders, C. Des Rochers, A. A. Lewis, M. H. Bennett

**Affiliations:** Cancer Research Campaign, Gray Laboratory, Middlesex, UK.

## Abstract

The proliferative potential of human solid tumours, in vivo, was investigated using bromodeoxyuridine (BrdUrd) incorporation and flow cytometry (FCM). Patients with solid tumours from a variety of sites were injected with 500 mg BrdUrd, intravenously, several hours prior to biopsy or surgical excision. The labelling index (LI), duration of S-phase (Ts) and thus the potential doubling time (Tpot) could be measured within 24 h of sampling. The results show that both the LI and Ts vary greatly between tumours (Ts ranges from 5.8 to 30.7 h). However, within this study of 26 evaluable patients, tumours of the same tissue origin tended to have similar Ts values. Melanomas had the shortest Ts (8.8 h), nine patients with head and neck cancer had Ts values ranging from 5.8 to 18.8 h (median 12.5 h). The longest Ts values (24 h) were found in lung and rectum. The estimates of Tpot ranged from only 3.2 days in an oat cell carcinoma to 23.2 days in a lymphoma. The striking feature of the study was that 38% of the tumours had a potential doubling time of 5 days or less. We found no relationship between proliferation and histopathological differentiation or DNA ploidy. It should now be possible to assess the prognostic significance of pretreatment cell kinetic measurements which may, in the future, aid in the selection of treatment schedules for the individual patient.


*Br. J. Cancer* (1988), **58**, 423–431
© The Macmillan Press Ltd., 1988

### Measurement of cell kinetics in human tumours *in vivo* using bromodeoxyuridine incorporation and flow cytometry

G.D. Wilson[1], N.J. McNally[1], S. Dische[2], M.I. Saunders[2], C. Des Rochers[2], A.A. Lewis[1] & M.H. Bennett[3]

[1]*Cancer Research Campaign, Gray Laboratory,* [2]*Marie Curie Research Wing; and* [3]*Department of Pathology, Mount Vernon Hospital, Northwood, Middlesex, HA6 2RN, UK.*

**Summary** The proliferative potential of human solid tumours, *in vivo*, was investigated using bromodeoxyuridine (BrdUrd) incorporation and flow cytometry (FCM). Patients with solid tumours from a variety of sites were injected with 500 mg BrdUrd, intravenously, several hours prior to biopsy or surgical excision. The labelling index (LI), duration of S-phase (Ts) and thus the potential doubling time (Tpot) could be measured within 24 h of sampling. The results show that both the LI and Ts vary greatly between tumours (Ts ranges from 5.8 to 30.7 h). However, within this study of 26 evaluable patients, tumours of the same tissue origin tended to have similar Ts values. Melanomas had the shortest Ts (8.8 h), nine patients with head and neck cancer had Ts values ranging from 5.8 to 18.8 h (median 12.5 h). The longest Ts values (24 h) were found in lung and rectum. The estimates of Tpot ranged from only 3.2 days in an oat cell carcinoma to 23.2 days in a lymphoma. The striking feature of the study was that 38% of the tumours had a potential doubling time of 5 days or less. We found no relationship between proliferation and histopathological differentiation or DNA ploidy. It should now be possible to assess the prognostic significance of pretreatment cell kinetic measurements which may, in the future, aid in the selection of treatment schedules for the individual patient.

The cellular proliferation of human cancer has been the subject of much study over the years, aimed at rationalising treatment so that schedules more suited to the cell kinetic characteristics of individual tumours can be given. However, progress has been hampered by the nature of the techniques available to measure cell kinetic parameters. The incidence of mitotic figures has been used to relate cell production rate to histological parameters and patient survival (Weiss, 1971). However, this parameter has failed to demonstrate any significant correlation with survival. The stathmokinetic method has been applied to human tumours by several groups (Meyer & Donaldson, 1969; Camplejohn *et al.*, 1973) to overcome the inadequacies of mitotic index alone. However, it is not possible to ensure that maximum mitotic collection rate is achieved. The most widely used method has been to measure the labelling index using tritiated thymidine ($^3$HTdR) and autoradiography. The bulk of the data obtained using this method have come from labelling tumour explants or cell suspensions *in vitro* (see Steel, 1977; Meyer, 1982 for reviews). Some studies have been performed *in vivo* (Frindel & Tubiana, 1968; Bennington, 1969; Young & DeVita, 1970; Terz *et al.*, 1971; Bresciani *et al.*, 1974). However, the technique suffers from two major drawbacks. Firstly, the result is not achieved in a time-scale suitable for the clinician to use if treatment is to be based on cell kinetic characteristics. Secondly, its wide use *in vivo* is precluded due to ethical considerations involved in administering a radioactive precursor of DNA and the requirement for multiple biopsies if cell cycle measurements are to be made.

A technique which allows cell kinetic measurements to be made on individual human tumours in a time-scale of use to the clinician in planning the most appropriate treatment, is that based on the flow cytometric measurement of bromodeoxyuridine (BrdUrd) incorporation into DNA (Gratzner, 1982; Dolbeare *et al.*, 1983). We have previously shown that it is possible to measure the labelling index (LI) of human tumours following an *in vivo* injection of BrdUrd, and using mouse tumours have shown that the BrdUrd technique gives the same information as the use of $^3$HTdR (Wilson *et al.*, 1985). The principal advantages of this flow cytometric technique are that it is rapid (the result can be obtained within a day of surgery), it can be used *in vivo* as BrdUrd is not radioactive or toxic at the doses required for cell kinetic studies, and it is possible to estimate both the LI and the duration of S-phase (Ts) and hence the potential doubling time (Tpot) from a single biopsy. The technique to estimate Tpot is based on the procedure first described by Begg *et al.* (1985), whereby a biopsy is taken several hours after an i.v. injection of BrdUrd.

In this study we describe results of measurements of Tpot in 26 patients with a variety of solid tumours and show that the method is applicable to routine measurements of cell kinetics in human tumours. In addition, we address the problem of heterogeneity in human tumours by studying multiple samples in four of the patients.

### Materials and methods

### *Patient selection*

Permission to administer BrdUrd was granted by the Ethical Committee of Mount Vernon Hospital and informed consent was obtained from each patient. The study included a spectrum of tumours, both primary and secondary, from the cervix, rectum, oesophagus, bronchus, head and neck and two melanomas (see Table I).

### *BrdUrd administration*

BrdUrd suitable for *in vivo* administration to humans was obtained from the Investigational Drug Division of the National Institute of Health, Bethesda, Maryland. Immediately prior to injection 500 mg BrdUrd was dissolved in 100 ml 0.9% saline and administered by intravenous infusion over 30 min (in our most recent studies the dose of BrdUrd has been reduced to 200 mg and is administered as a single push in 20 ml normal saline). A biopsy or surgical excision was performed in each case between 4 and 22 h after injection (most were taken between 4 and 8 h). No toxicity was observed in any patient given 500 mg BrdUrd in this manner. In all cases an adjacent portion of material was examined histologically so that the proportion of the specimen occupied by tumour cells, stroma and necrosis could be estimated.

### *Cell preparation*

The tumours were processed into single cells by a variety of

Correspondence: G.D. Wilson.
Received 16 March 1988; and in revised form, 7 June 1988.

methods depending on the nature of the material. The procedures have been described in detail elsewhere (Wilson *et al.*, 1985). In general, dissociation by mechanical means was always attempted first. If this proved unsuccessful, the tumour fragments were incubated in 0.2% collagenase (type II, Sigma Chemical Co., Poole, England) and 0.02% DNAse I (Sigma Chemical Co.) in Hanks Balanced Salt Solution without calcium or magnesium. More recently, tumours have been digested with $0.4\,mg\,ml^{-1}$ pepsin in 0.1 M HCl for 1 h at 37°C to release nuclei after prior fixation of solid pieces in 70% ethanol (Schutte *et al.*, 1987).

*BrdUrd staining and flow cytometry*

The procedures for BrdUrd staining and FCM analysis have been described in detail previously (Wilson *et al.*, 1985). Several techniques for DNA denaturation were tested to unmask BrdUrd binding sites. The most successful technique was incubation at room temperature with 2 M HCl for 15 to 30 min. Procedures involving heat denaturation resulted in an unacceptable amount of cell loss and clumping. The anti-BrdUrd antibody used in this study was a rat monoclonal antibody supplied by Dr. M. Ormerod of the Institute of Cancer Research, Sutton. The antibody was incubated with the cells for 1 h at room temperature at a dilution of 1:25. Incubation with the second FITC-labelled antibody was for 30 min and cells were analysed 15 min after addition of $10\,\mu g\,ml^{-1}$ propidium iodide (PI) in 3 ml PBS. Stained preparations were analysed by an Ortho Systems 50-H Cytofluorograph with excitation at 488 nm (100 mW) from a 5 W argon ion laser. Green fluorescence (FITC) was collected between 510–560 nm and red fluorescence (PI) above 620 nm. Data were collected in list mode and cell doublets, triplets etc., were excluded from the analysis by gating on the DNA peak versus area signal. 10,000 to 50,000 cells were analysed for each specimen.

### Results

Table I shows the clinical and histopathological details of the patients involved in this study. The series included a spectrum of both primary, recurrent and metastatic tumours from different sites. Fourteen of the tumours were squamous cell carcinomas and 6 were adenocarcinomas. The largest series from an individual site were nine head and neck tumours (group E).

Figures 1 and 2 show examples of the staining profiles obtained several hours after administration of BrdUrd to patients with solid tumours. Figure 1A shows the DNA profile and 1B the bivariate cytogram of DNA content *versus* BrdUrd uptake for a moderately differentiated adenocarcinoma of the rectum removed 5.5 h after injection of BrdUrd (patient C1 in Table I). The tumour was aneuploid (Figure 1A) with a DNA index of 1.6. The hyperdiploid component (i.e. those cells with greater than 2n DNA content) represented 73% of the total cell population. The bivariate cytogram (Figure 1B) shows relative green (BrdUrd) *versus* red (DNA) fluorescence on a linear scale. In tumours such as this it can readily be seen that the proliferative cells are associated with the aneuploid population. The LI is defined as the total number of cells in the population which are synthesising DNA and can be measured by setting a region around those cells which show significant BrdUrd incorporation. The lower limit of this region delineating BrdUrd labelled cells was determined by a parallel incubation of the cell suspension with the omission of the anti-BrdUrd monoclonal antibody. In this case the total LI was 13.7%. An estimate of the aneuploid LI can be made by setting two regions close to the left edge of the aneuploid G1 population, one around the total number of aneuploid cells and the other around those cells which show significant BrdUrd uptake. The LI of the aneuploid cells, in this tumour, was 17.5%. However, the BrdUrd labelled cells associated with the aneuploid component are now distributed into two populations. The majority of labelled cells, as denoted by the rectangular region, are still progressing through S-phase but show a distribution skewed towards G2+M. They are clearly separated from BrdUrd labelled cells which have divided and are now in G1 and which are distinguished from other G1 cells by virtue of their BrdUrd uptake at the time of injection. The division of BrdUrd labelled cells in the period between injection and biopsy creates an error in the estimation of the LI as the DNA precursor will be shared between the two daughter cells at division. This will lead to an overestimation of the LI which will increase as the time period between injection and biopsy increases. The simplest correction would be to calculate the

**Table I** Clinical details of patients given with BrdUrd for cell kinetic studies

| *Code* | *Site of primary* | *Site biopsied* | *Histology* |
|---|---|---|---|
| B1 | Cervix | Primary | Well diff, SCC |
| B2 | Cervix | Primary | Anaplastic small cell |
| B3 | Cervix | Primary | Anaplastic SCC |
| C1 | Rectum | Recurrent primary | Moderately diff adenoca. |
| C2 | Rectum | Recurrent primary | Papillary well diff adenoca. |
| C3 | Rectum | Recurrent primary | Moderately diff adenoca. |
| C4 | Rectum | Recurrent primary | Moderately diff adenoca. |
| D1 | Oesophagus | Skin metastasis | Moderately diff adenoca. |
| D2 | Oesophagus | Primary | Moderate to poor diff adenoca. |
| D3 | Oesophagus | Primary | Poorly diff SCC |
| D4 | Oesophagus | Recurrent primary | Poorly diff SCC |
| E1 | Larynx | Skin metastasis | Poorly diif SCC |
| E2 | Tongue | Primary | Very well diff SCC, verrucal. |
| E3 | Nose/lip | Recurrent primary | Well diff SCC |
| E4 | Larynx | Primary | Hyperkeratotic papilliferous SCC |
| E5 | Columella | Primary | Well diff keratinising SCC |
| E6 | Retromolar | Primary | Moderately diff SCC |
| E7 | Retromolar | Primary | Well diff SCC |
| E8 | Tongue | Primary | Moderate diff keratinising SCC |
| E9 | Tongue | Recurrent primary | Moderately diff SCC. |
| F1 | Bronchus | Skin metastasis | Oat cell ca. |
| F2 | Bronchus | Primary | Poorly diff SCC, keratinising |
| F3 | Bronchus | Primary | Oat cell ca. |
| H1 | Supraclavicular | Recurrent primary | Diffuse non-Hodgkins lymphoma |
| I1 | Skin | Skin metastasis | Malignant melanoma |
| I2 | Skin | Skin metastasis | Malignant melanoma |

**Figure 1** DNA profile (a) and bivariate cytogram (b) of BrdUrd uptake *versus* DNA of patient C1. The tumour was a moderately differentiated adenocarcinoma of the rectum. The biopsy was removed 5.5 h after BrdUrd administration. The DNA profile was aneuploid with an index of 1.6. The region in (b) denotes the population of cells still progressing through the cell cycle towards G2+M from which the Ts measurement is made.

**Figure 2** DNA profile (a) and bivariate cytogram (b) of BrdUrd uptake *versus* DNA of patient I1. The tumour was a malignant melanoma. A skin nodule was removed 6.5 h after BrdUrd administration. It is evident from (b) that the majority of labelled cells have divided and are now in G1.

number of BrdUrd labelled cells which had divided (both in the diploid and the aneuploid populations) and subtract half of this number from the total number of labelled cells and from the total number of cells. This, however, may lead to an underestimation of the true LI as it does not take into account unlabelled G2 cells which will also have divided and expanded the cell population at the time of biopsy. If it were also possible to estimate the number of G2 cells which had divided then a better estimate of the true LI could be made. However this is not possible because the unlabelled cells in G1 at the time of biopsy will be a mixture of cells that were in G1 at the time of labelling that have not yet progressed into S and G2 cells that have divided. Consequently, we have corrected the LI simply by halving the number of labelled cells which have divided. This correction reduces the LI to 12.4 and 16.0% in the total cell and hyperdiploid only populations, respectively, in Figure 1B. The estimation of Ts from profiles such as this was made using the technique described by Begg *et al.* (1985). In this analysis the mean DNA content of the BrdUrd labelled cells still progressing through S-phase (i.e. those cells in the rectangular region) is expressed as a proportion of the difference in DNA content between G1 and G2+M cells. This is achieved by measuring the mean DNA content of G1 and G2+M cells either from the bivariate cytogram or the DNA profile and the mean DNA content of the BrdUrd labelled cells which are still progressing through S and have not yet divided. The 'relative movement' (R.M.) is then calculated by subtracting the mean DNA of G1 cells from that of the S-phase population and dividing it by the G1 DNA content subtracted from the G2 DNA content. In order to make the calculation of Ts from the R.M., three assumptions are made: firstly, that immediately after injection with BrdUrd, the labelled S-phase cells are evenly distributed throughout S-phase with a mean DNA content in mid-S, giving a relative movement of 0.5; secondly, that the R.M. reaches 1.0 when those labelled cells that had just entered S-phase reach G2; i.e. in a time equal to Ts; thirdly, that the R.M. is a linear function of time. It is then possible to calculate Ts from a single measurement of R.M. at some time after pulse labelling with BrdUrd. In Figure 1B the R.M. was 0.61 giving a Ts of 25.8 h. Using this value and the LI of 16.0%, the potential doubling time (Tpot) can be calculated from the relationship; Tpot = $\lambda$. Ts/LI, where $\lambda$ is a correction factor for the age distribution of the population. A value of 0.8 was used in these calculations (see Steel, 1977), giving a Tpot of 5.4 days in this tumour.

Figure 2 shows the profiles obtained from a malignant melanoma biopsied 7 h after BrdUrd injection (Patient I1 in Table I). The DNA profile (Figure 2A) shows that the cell population was diploid. The striking feature of this specimen was that the majority of BrdUrd labelled cells had divided and entered G1 in 7 h. The Ts measurement gave a value of 8.8 h and this, in conjunction with a corrected LI of 8.3%, gave a Tpot of 3.2 days. This is extremely short, implying very rapid proliferation in this tumour.

Table II summarizes the cell kinetic data we have obtained in the series of 26 evaluable patients using this technique. Fourteen of the patients (54%) had aneuploid tumours. The numbers are too small to assess whether there is a significant relationship between differentiation and DNA ploidy in this series. However, Figure 3A shows that of 14 squamous cell carcinomas from various sites only 1 of 5 well differentiated tumours was aneuploid whilst only 1 of 8 moderate or poorly differentiated tumours was diploid. There was no relationship between differentiation and potential doubling time in this group of tumours (Figure 3B). In 6 adenocarcinomas, the only well differentiated tumour was diploid and the only poorly differentiated tumour was aneuploid.

The total cell population LI of all tumours in this study ranged from 2.3 to 20.6% with a median value of 6.4%. Eight tumours (31%) had a total LI of 10% or greater; 4 were aneuploid and 4 diploid. The median total LI of diploid tumours (n = 12) was 8.3% compared to 5.7% in the aneuploid tumours (n = 14). The median LI in aneuploid tumours was increased to 10.0% when the measurement was made on the hyperdiploid population only. Using this refinement, the number of tumours with LIs of 10% or greater increased to 11 (42%), and of these 7 were aneuploid and 4 diploid.

The Ts measurements in this series of patients ranged from 5.8 h to 30.7 h, the median value being 16.2 h. Five tumours had a Ts of less than 10 h. Three of these were head and neck tumours whilst the other two were melanomas. Figure 4 shows the mean Ts values for each of the groups of tumours. Although the numbers are quite small in each group of tumours apart from the head and neck patients, there appears to be a trend for tumours of the same origin to have a similar Ts. Consequently, Figure 4 shows the mean rather than median value of Ts together with the s.e.m. (solid bars) and range (dotted bars) to give an indication of the variation in this parameter between different groups of tumours. Figure 4 shows that there are striking differences in Ts from tumours of different sites. There is a gradation in Ts throughout the upper aerodigestive tract. Head and neck tumours have a short DNA synthetic period (mean 11.8 ± 1.2 h). Tumours from oesophageal sites all had very similar Ts values (mean 16.5 ± 0.4 h) whilst lung tumours had longer S-phases (24.3 ± 2.7 h). Rectal tumours tended to have a long Ts (24.5 ± 2.9 h) whilst the two melanomas had the shortest Ts values (8.8 and 8.7 h).

The calculated potential doubling times ranged from 3.2 days in an oat cell carcinoma (F1) to 23.2 days in a lymphoma (H1). The median Tpot was surprisingly short at 5.6 days. Ten tumours (38%) had a Tpot of less than 5 days; of these 7 were aneuploid and 3 diploid. Figure 5A shows the relationship between total LI and measured Tpot. Dotted lines have been drawn through a LI of 10% and a Tpot of 5 days. These are 'threshold' values, below which patients may benefit from a reduction in overall treatment time (Fowler, 1985, Thames *et al.*, 1983). If the LI were the only parameter measured, as was the case in previous studies with $^3$HTdR,

**Table II** Cell kinetic parameters of patients injected with BrdUrd *in vivo*. The aneuploid LI was calculated by excluding cells with 2n DNA content (both BrdUrd labelled and non-labelled cells). The Tpot measurements are calculated using the total LI in diploid tumours and the aneuploid population LI in aneuploid tumours.

| *Code* | *DNA index* | *Total LI (%)* | *Aneuploid LI (%)* | *Ts (hr)* | *Tpot (days)* |
|---|---|---|---|---|---|
| B1 | 2.4 | 15.2 | 17.0 | 22.0 | 4.3 |
| B2 | 2.1 | 6.6 | 15.7 | 20.8 | 4.4 |
| B3 | 1.6 | 5.6 | 11.0 | 11.8 | 3.6 |
| C1 | 1.6 | 12.4 | 16.0 | 25.8 | 5.4 |
| C2 | 1.0 | 10.3 | | 30.7 | 9.9 |
| C3 | 1.0 | 14.7 | | 25.0 | 5.7 |
| C4 | 1.0 | 10.7 | | 16.6 | 5.2 |
| D1 | 1.3 | 5.5 | 14.2 | 15.6 | 3.7 |
| D2 | 1.7 | 5.8 | 7.6 | 16.7 | 7.3 |
| D3 | 2.6 | 2.5 | 6.4 | 17.5 | 9.1 |
| D4 | 1.8 | 6.1 | 8.0 | 16.3 | 6.8 |
| E1 | 1.0 | 8.3 | | 18.8 | 7.6 |
| E2 | 1.0 | 2.9 | | 5.8 | 6.7 |
| E3 | 1.0 | 8.9 | | 13.3 | 5.0 |
| E4 | 1.0 | 7.8 | | 9.5 | 4.1 |
| E5 | 1.0 | 3.4 | | 12.5 | 12.3 |
| E6 | 1.5 | 12.8 | 13.2 | 13.3 | 3.4 |
| E7 | 1.0 | 4.6 | | 10.9 | 7.9 |
| E8 | 2.0 | 5.0 | 9.1 | 12.5 | 4.6 |
| E9 | 1.4 | 3.2 | 7.3 | 9.7 | 4.4 |
| F1 | 1.0 | 20.6 | | 20.0 | 3.2 |
| F2 | 1.6 | 4.3 | 13.8 | 29.4 | 11.3 |
| F3 | 1.2 | 11.9 | 14.5 | 23.4 | 5.7 |
| H1 | 1.0 | 2.3 | | 16.0 | 23.2 |
| I1 | 1.0 | 8.3 | | 8.8 | 3.5 |
| I2 | 1.7 | 3.4 | 4.2 | 8.7 | 6.9 |

**Figure 3** Relationship between histological assessment of differentiation and DNA ploidy (a) or Tpot (b) in 13 squamous cell carcinomas from a variety of different sites.

**Figure 4** Ts measurements of tumours from different sites. The results are presented as mean ±s.e.m. (solid lines) and range (dotted line) for each tissue.

then 13 patients would have been wrongly classified into the 'rapid' or 'slow' proliferating groups. Eight of these were patients whose tumours had LIs less than 10% but Tpots of less than 5 days; the other five had high LIs but longer Tpots. Figure 5B shows that the situation was only slightly improved when the aneuploid LI was used, where it was measurable. Five patients remained in the low LI short Tpot group and 5 in the higher LI long Tpot group. All 4 cases of rectal carcinoma fell into the group of high LI but long Tpot whilst 4 cases of head and neck tumours were found in the low LI short Tpot group. This demonstrates the dependence of Tpot on Ts.

Any procedure which is based upon measurements from a single small biopsy suffers from the criticism that the biopsy may not be representative of the whole tumour. We have addressed this problem in four of the tumours, in which it was possible to take several samples. Figure 6 shows the results obtained for patient B3. The specimens consisted of four samples selected at random by the pathologist from a large surgically resected tumour of the cervix; histologically the four pieces all contained anaplastic squamous carcinoma cells but there were differences in the amount of stromal and of necrotic areas. Figure 6a shows the relative amounts of tumour, stroma and necrosis in each sample, estimated histologically, and should be compared with Figure 6b in which the percentage of 'tumour' cells (i.e., those with greater than 2n DNA content) has been measured by FCM analysis of the DNA profile. Qualitatively the variation in the presence of aneuploid cells matched the histological assessment of the amount of tumour in each sample. Sample 2 had the least tumour histologically and the smallest fraction of aneuploid cells. The variation in the presence of aneuploid cells between samples is reflected in the variation in total LI (Figure 6c). This ranged from 4.3 to 7.3% with a mean of 5.6±0.7%. The aneuploid LI (calculated excluding

**Figure 5** Correlation between LI and the measured Tpot. The total cell population LI, of both diploid and aneuploid tumours, is shown in (a). In (b) the LI of the 14 aneuploid tumours (solid symbols) has been calculated excluding the normal G1; the LI of the 12 diploid tumours (open symbols) remains as in (a). The dotted lines represent the cut-off denoting 'rapidly' or 'slowly' proliferating tumours for each parameter.

**Figure 6** Heterogeneity in cell kinetic parameters from a patient with an anaplastic squamous cell carcinoma of the cervix (b3). The histograms represent individual values for histological assessment of percent of tumour □, stroma ▧ and necrosis ■ (a), % of hyperdiploid cells (b), total LI (c), aneuploid LI (d), Ts (e) and Tpot (f) obtained from each of 4 samples.

the diploid G1) showed less variation (Figure 6d), with a mean of 11.0±0.9%. There was very little difference in the estimate of Ts between the samples (range 10.5 to 12 h) (Figure 6e) with the result that the variation in the calculated potential doubling time reflected, primarily, variations in the LI between samples. This ranged from 2.9 to 4.6 days with a mean of 3.6±0.4 days (Figure 6f). There was no change in the DNA index between the samples (results not shown).

Figure 7 shows results obtained in patient C4. This patient had previously undergone a resection and anastomosis for carcinoma of the rectum. The specimens removed for this study came from a recurrence in the rectal stump. Four areas of the tumour were sampled as was a polypoid fragment. All samples, including the tissue from the polypoid region, showed similar histology with presence of a moderately differentiated and partly mucin secreting adenocarcinoma similar to the primary. The DNA content of the specimens was diploid. In this patient, there was variation in both the LI (Figure 7a) and Ts (Figure 7b) and, hence, the Tpot (Figure 7c) measurements between the individual samples. The LI ranged from 8.9 to 13.1% and the Ts from 13 to 19 h in the biopsies from the main tumour area which resulted in a range of Tpot of 3.4 to 6.9 days. The cell kinetic characteristics of the tumour tissue from the polypoid area was markedly different to the other samples; the LI was 8.7% and the Ts 31.9 h giving a Tpot of 12.2 days.

The other two patients, D4 and H1, did not show any significant difference in cell kinetic parameters between different biopsies. Six biopsies were processed from patient H1; all 6 showed similar histology with the presence of diffuse grade II non-Hodgkin's lymphoma. The tumour was diploid and the LI and Ts ranged from 2.2 to 2.5% and 14.7 to 16.7 h, respectively. The estimates of Tpot ranged from 21.3 to 25.3 days (Figure 8). Patient D4, had a poorly differentiated infiltrating SCC of the oesophagus which extended to the surrounding connective tissue. Two areas were studied, one sample was taken from the lumen surface and the other from the area infiltrating the connective tissue; these specimens showed similar histology. The tumour was

**Figure 7** Heterogeneity in cell kinetic parameters from a patient with a moderately differentiated adenocarcinoma of the rectum (C4). The histograms represent individual values of LI (a), Ts (b) and Tpot (c) obtained from 4 areas of the tumour and a polypoid fragment.

**Figure 8** Heterogeneity in cell kinetic parameters from a patient with a diffuse non-Hodgkin's lymphoma (H1). The histograms represent individual values of LI (a), Ts (b) and Tpot (c) from six areas of the tumour.

aneuploid, but there were no significant differences in DNA index, % of hyperdiploid cells, total LI, aneuploid LI, Ts or Tpot (Figure 9).

### Discussion

This study demonstrates the feasibility of making routine measurements of cell kinetics in solid human tumours using BrdUrd administration and FCM. The technique offers obvious advantages over previous techniques using $^3$HTdR and autoradiography. Labelling can be done *in vivo* without the problems associated with the administration of radioactive material to patients or multiple sampling. BrdUrd is non-toxic at the dose (500 mg) used in these studies, and we have recently reduced this dose to 200 mg without any loss of resolution of the BrdUrd profiles; doses as high as 1,000 mg m$^{-2}$ day$^{-1}$ for up to 2 weeks have been administered to patients in radiosensitization studies with no acute toxicity (Kinsella *et al.*, 1984). In addition, the results can be obtained within a day.

Ten patients who were administered BrdUrd failed to give a satisfactory result (data not shown). However, in 7 of the cases that failed there were too few viable cells in the biopsy either because of extensive necrosis or the biopsy was too small to give sufficient cells for FCM (at least 1 million cells at the start of staining are required). There were 3 cases in which the BrdUrd staining failed for no apparent reason.

The parameters that can be measured from a single biopsy are the LI, Ts and thus Tpot. The potential doubling time is the doubling time of a population of cells taking into account the growth fraction but ignoring cell loss. There is evidence in both animal and human tumours that repopulation rates during or after radiotherapy are similar to the potential doubling time. Maciejewski *et al.* (1983) showed that local control of T3/4 larynx cancer fell from acceptable levels when the overall treatment time was extended beyond 6 weeks. The dose to control 50% of the tumours increased by about 0.5 Gy per day which is consistent with a population doubling time of about 4 days. This is similar to

**Figure 9** Heterogeneity in cell kinetic parameters from a patient with a poorly differentiated SCC of the oesophagus (D4). The histograms represent individual values of % of hyperdiploid cells (a), total LI (b), hyperdiploid LI (c), Ts (d), Tpot (e) and DNA index (f) from an area abutting the lumen of the bronchus (1) and an area infiltrating the connective tissue (2).

**Figure 10** Errors involved in LI (a) and Tpot (b) if correction for division of BrdUrd labelled cells between injection and biopsy is not used.

the potential doubling time of the untreated tumours calculated from the average $^3$HTdR LI of 15% observed in squamous cell carcinomas of the head and neck, assuming a Ts of 18 h (Trott & Kummermehr, 1985). Parsons *et al.* (1980) showed that local control and 5 year survival, for patients with head and neck cancer, improved if 30 fractions were given in 6 weeks compared to 30 fractions in 8 weeks with a two week gap in the middle. An average improvement of 16% was seen in local control for all stages of disease. This represents a 10% increase in effective dose and an increment of 1 log of cell kill. This corresponds to at least 3 doublings of cell number in 2 weeks (Fowler, 1985). This doubling time is close to the value of Tpot mentioned above. An estimate of repopulation rates of human tumours can be made from the latency time to local recurrence after potentially curative radiotherapy (Thames *et al.*, 1983), assuming that subsequent growth of uncontrolled tumours starts from a few surviving clonogenic cells. Using this approach, Trott & Kummermehr (1985) showed that the tumour groups with the longest Tpots (measured from *in vitro* $^3$HTdR LIs and assuming a Ts of 18 h) had the longest latency period to local recurrence and vice-versa for the short Tpots.

The cell kinetic measurements made in this study agree with the limited *in vivo* data available in the literature. Most studies utilising the percent labelled mitosis curve technique were reviewed by Steel (1977). From a variety of different sites, excluding ascitic and pleural effusions, the average Ts was 18.1 h and ranged from 11 h in a carcinoma of the maxillary antrum (Terz *et al.*, 1971) to 25 h in a melanoma (Shirakawa *et al.*, 1970) and a neuroblastoma (Wagner & Kaser, 1972). In many of these studies the criterion for patient selection meant that very advanced stage disease was selected. There has been a report by Japanese workers concerned with measuring Ts in solid tumours by an *in vitro* double labelling technique with high and low specific activity $^3$HTdR. Sakuma (1980) measured Ts values ranging from 4.1 to 14.0 h in human squamous cell carcinomas of the oral cavity. This range of values is very similar to the observations made in our study with head and neck tumours. We have not attempted this technique in solid tumours but in collaboration with Riccardi *et al.* (1988), we have shown that in 13 cases of haematological disease where Ts was measured simultaneously by the double labelling technique of Dormer (1973) and the BrdUrd/DNA staining method the two estimates were similar (correlation coefficient 0.83, $P \leq 0.05$). In that study there was also a good correlation between BrdUrd LI and $^3$HTdR LI measured simultaneously in 48 patients with haematological tumours or normal bone marrow ($r = 0.91$, $P \leq 0.05$).

It is difficult to assess if LI measurements *in vivo* agree with those obtained from *in vitro* techniques because of patient selection procedures. Our previous study (Wilson *et al.*, 1985) using *in vitro* BrdUrd incorporation, and unpublished observations, showed a median total LI of 5.0% in 26 cases of colorectal cancer whilst in four cases *in vivo* in this present study, the median was 11.6%. In 75 measurements from all tumour types, *in vitro*, the overall median LI was 5.3% whilst the median LI in this present *in vivo* study of 26 patients was 6.4%. Any differences could reflect shortcomings in achieving complete incorporation of DNA precursors into tumour fragments *in vitro*. Differences were observed, to some extent, in a study by Chavaudra *et al.* (1979). These authors measured LI *in vivo* and *in vitro* simultaneously in 16 human solid squamous cell carcinomas of the head and neck using either incubation with or injection of $^3$HTdR. The mean LI *in vivo* was 16.3% whilst that *in vitro* was only 11.5%. They showed that in 10 of the tumours there was reasonable agreement between the two methods but that there was serious underestimation of LI by *in vitro* labelling in the other 6 tumours. The overall conclusion, from their study, was that *in vitro* techniques may lead to an underestimation of LI due either to heterogeneity from one biopsy to another in the same tumour or a lower uptake of DNA precursor in the deep cell layers of the *in vitro* labelled tumour fragments. If heterogeneity was the problem, then one might have expected the variation between *in vivo* and *in vitro* LI to have been more random. This data and our own data suggests that *in vitro* labelling is underestimating the true labelling index. This study and our previous study (Wilson *et al.*, 1985) have demonstrated the presence of a significant proportion of S-phase cells which do not appear to take up BrdUrd. In this study we are not able to estimate the relative proportions of these two populations as we are delaying the biopsy after injection such that at the time we make our observation there will be cells in S-phase which do not show BrdUrd uptake because they were in G1 at the time of the injection. It is apparent that this population of S-phase cells varies from tumour to tumour and can be as small as only a few percent of the total S-phase population or can be as great as 50%. The identity and significance of these cells has yet to be established. The lack of BrdUrd uptake might be due to failure to be exposed to the DNA precursor or to arrest or slowing down of DNA synthesis due to changing microenvironments. There has been recent evidence (Chaplin *et al.*, 1987) to suggest that there may be transient opening and closing of blood vessels in tumours leading to foci of hypoxia. Such a mechanism could result in our observation of unlabelled S-phase cells as some cells may not see the BrdUrd as vessels around them were closed at the time of injection or because they had become temporarily arrested due to hypoxia. The question then arises whether these unlabelled cells are functional or could become functional.

We have calculated the potential doubling time according to the simple procedure described by Begg *et al.* (1985) with the assumptions as indicated above. In spite of the more detailed mathematical considerations in the appendix of the paper by Begg *et al.* (1985) and by White & Meistrich (1986) we have found the assumption of linearity in the change in R.M. with time to be a reasonable one in cells in culture, in mouse bone marrow, in cells of the mouse kidney and in two transplantable mouse tumours (Begg *et al.*, 1985, Wilson

*et al.*, 1987; Wilson & McNally, unpublished observations). In all but one of these cases the initial value of the R.M. was not significantly different from 0.5. For one tumour (SAF) it was closer to 0.4. Of course it has not been possible to make comparable measurements in human tumours.

A potential source of error in measuring the LI arises through cell division in the time between injecting the BrdUrd and taking the biopsy. The larger this time, the greater the error will be. We have made the simplest, and probably only technically feasible, correction of halving the number of BrdUrd labelled cells that appear in G1, as described above. Figure 10 shows the effect of not making this correction on both the LI (A) and Tpot (B). The number of tumours with a LI of greater than 10%, increased from 12 to 17 if the correction was not made and the number of with a Tpot of 5 days or less increased from 11 to 14. The greatest effect of not making the correction was in those tumours with the longest interval between injection and biopsy and in those which had a very short Ts. If the time interval is too short, however, it may be difficult to detect a significant movement of BrdUrd labelled cells towards G2. Based on these limited results, we feel that intervals of 3 to 4 h for melanomas and head and neck tumours, and between 6 to 8 h for other tumour types may be most suitable.

The technique we have used to measure Tpot is based on measurements from a single biopsy. It is important, therefore, to try and assess the potential error due to heterogeneity within individual tumours with respect to their cell kinetics. We have addressed this problem in four of the patients we have studied. There were no differences in the cell kinetic parameters measured in six samples from a non-Hodgkin's lymphoma (Figure 8) or two biopsies from a SCC of the oesophagus (Figure 9). There were some variations in an aneuploid SCC of the cervix (patient B3, Figure 6). The greatest variation was in the total LI which varied from 4.3 to 7.3%. However, this was largely a reflection of variations in the extent of normal stromal tissue in each sample which were seen histologically and in the flow cytometer as variations in the fraction of the population that was hyperdiploid (Figure 6b). When the potential doubling time was calculated excluding the 'normal' G1 cells, there was relatively little variation from one sample to the next (mean Tpot 3.6 days, range 2.9 to 4.6 days, Figure 6f). This example illustrates the importance of having parallel histology of tissue adjacent to pieces processed for FCM. Indeed, not only can the FCM analysis be related to the histology, but BrdUrd incorporation can also be assessed in the tissue section by immunohistochemical staining (Wilson *et al.*, 1988). In these three cases the main differences in cell kinetics from one sample to the next were in the LI with relatively small differences in the estimate of Ts. However, in the five samples from the recurrent carcinoma of the rectum (patient C4, Figure 7), there was variation in both LI and Ts. In particular, the tissue taken from the polypoid fragment showed a much longer Ts (30.9 h) than the other four samples from the main bulk of the tumour which ranged from 13.2 to 19.3 h. The reason for this is unclear as there was no histological difference in the tumour from the two areas. These differences, in conjunction with variation in the LI (range 8.7 to 13.1%), resulted in a large range of Tpot throughout this tumour (3.4 to 9.8 days). Although parallel histology was not processed for each sample, the specimen was moderately differentiated and consisted of approximately 50% tumour cells and 50% normal stromal cells. It is possible that there were differences in the pattern of cellularity of each sample which could account for the observed differences.

It would appear, from these four examples, that the principal source of variation between different samples is the LI. This, in turn, is reflected in relatively small variations in the estimated Tpot in all but one of the cases we have studied. Certainly, from a predictive point of view the variation we have measured would not be serious. If, for instance, patients were going to be selected for accelerated fractionation based on their kinetic parameters, one would not need to have a sharp delineation in the value of Tpot below which patients would be given the altered treatment and above which they would not. If a Tpot of 3.4 days was estimated and the true value was 7 days, the selection for accelerated fractionation would almost certainly not compromise the outcome (and *vice versa*).

The estimates of Tpot in this group of patients are striking because of the proportion of tumours which had the potential for rapid proliferation. Thirty-eight percent of the tumours in this study (from all the sites) had Tpots of less than 5 days. The shortest Tpot of 3.2 days was seen in a patient with metastatic oat cell carcinoma. Seven of the 16 patients with tumours of the head and neck, oesophagus or bronchus, sites accessible for biopsy and suitable for accelerated fractionation, had Tpots of less than 5 days. These estimates of Tpot are similar to the few that can be computed from the data collected by Steel (1977).

Using the median Ts, the LI and a correction factor of 0.8, then the cases reported by Steel ranged from 1.3 days in a tumour of the maxillary antrum (Terz *et al.*, 1971) to 11.7 days in a reticulum cell sarcoma (Peckham & Steel, 1972). The study by Sakuma (1980) reports very short Tpots, in carcinoma of the oral cavity, ranging from 0.7 to 2 days. In radiotherapy, it has been calculated that if overall treatment times of 6 or 7 weeks are shortened to one half or one third by using 2 or 3 fractions per day then gains in local control can be achieved if the doubling time of clonogenic tumour cells is 5 days or shorter (Thames *et al.*, 1983). The rationale behind this approach is that if tumours are repopulating rapidly, then reducing the overall treatment, for instance by 21 days, will save 4.2 cell doublings in tumours with clonogenic doubling times of 5 days or less. This corresponds to a factor of 18 decrease in the number of cells which have to be killed by the treatment.

For patients to benefit from an altered treatment schedule, such as accelerated fractionation, it is important that cell kinetic information can be obtained with the minimum inconvenience to the patient and in a timescale useful to the clinician in selecting the appropriate treatment. The technique described here can obtain the relevant information within a day of biopsy without undue stress to the patient. The value of pretreatment cell kinetic measurements has yet to be verified in radiotherapy. Accelerated fractionation employing 3 treatments each day for a continuous period of 12 days is currently being employed at Mount Vernon Hospital to treat advanced head and neck and bronchial tumours (Saunders & Dische, 1986) with favourable regression and remission rates compared to previous studies. Whenever possible, patients receiving this treatment are now having their pretreatment cell kinetics measured. In future randomised controlled trials of accelerated fractionation, measurements of Tpot as we have described should be included in both arms of the trial. If it is shown that tumours with short potential doubling times are best treated in an accelerated course of radiotherapy, then there will be important implications, not only for the patients receiving radiotherapy but, also for those being managed by cytotoxic chemotherapy where rapid repopulation between cycles of treatment would have a similarly harmful influence upon tumour control, and measurements of Tpot should become part of the routine characterisation of the tumour before treatment.

This work was supported by the Cancer Research Campaign. We thank Professor J.F. Fowler for helpful discussions. We are grateful to the surgeons at Mount Vernon Hospital and Mr E. Townsend at Harefield Hospital for their co-operation with this work.

### References

BEGG, A.C., McNALLY, N.J., SHRIEVE, D.C. & KARCHER, H. (1985). A method to measure the duration of DNA synthesis and the potential doubling time from a single sample. *Cytometery*, **6**, 620.

BENNINGTON, J.L. (1969). Cellular kinetics of invasive squamous cell carcinoma. *Cancer Res.*, **28**, 2187.

BRESCIANI, F., PAOLUZI, R., BENASSI, M., NERVI, C., CASALE, C. & ZIPARO, E. (1974). Cell kinetics and growth of squamous cell carcinomas in man. *Cancer Res.*, **34**, 2405.

CAMPLEJOHN, R. S., BONE, G. & AHERNE, W. (1973). Cell proliferation in rectal carcinoma and rectal mucosa, a stathmokinetic study. *Eur. J. Cancer*, **9**, 577.

CHAPLIN, D.J., OLIVE, P.L. & DURAND, R.E. (1987). Intermittent blood flow in a murine tumour: Radiobiological effects. *Cancer Res.*, **47**, 597.

CHAVAUDRA, N., RICHARD, J.M. & MALAISE, E.P. (1979). Labelling index of human squamous cell carcinomas. *Cell Tissue Kinet.*, **12**, 145.

DOLBEARE, F.A., GRATZNER, H.G., PALLAVICINI, M.G. & GRAY, J.W. (1983). Flow cytometric measurement of total DNA content and incorporated bromodeoxyuridine. *Proc. Natl Acad. Sci.*, **80**, 5573.

DORMER, P. (1973). Kinetics of erythropoietic cell proliferation in normal and anemic man. A new approach using quantitative $^{14}$C-autoradiography. *Prog. Histochem. Cytochem.*, **6**, 1.

FOWLER, J.F. (1985). Potential for increasing the differential response between tumours and normal tissues: Can proliferation rate be used? *Int. J. Radiat. Oncol. Biol. Phys.*, **12**, 641.

FRINDEL, E. & TUBIANA, M. (1968). Cell proliferation kinetics in five human solid tumouirs. *Cancer*, **22**, 611.

GRATZNER, H.G. (1982). Monoclonal antibody to 5-bromo- and 5-iododeoxyuridine: a new reagent for detection of DNA replication. *Science*, **218**, 474.

KINSELLA, T.J., RUSSO, A., MITCHELL, J.B. & 10 others (1984). A phase I study of intermittent intravenous bromodeoxyuridine (BUdR) with conventional fractionated irradiation. *Int. J. Oncol. Biol. Physiol.*, **10**, 69.

MACIEJEWSKI, B., PREUSS-BAYER, G. & TROTT, K.-R. (1983). The influence of the number of fractions and of overall treatment time on local control and late complication rate in squamous cell carcinoma of the larynx. *Int. J. Radiat. Oncol. Biol. Phys.*, **9**, 321.

MEYER, J.S. (1982). Cell kinetic measurements of human tumours. *Human Path.*, **13**, 874.

MEYER, J.S. & DONALDSON, R.C. (1969). Growth kinetics of squamous cell carcinoma in man. *Archs. Path.*, **87**, 479.

PARSONS, J., BOVA, F.J. & MILLION, R.R. (1980). A re-evaluation of split-course technique for squamous cell carcinoma of the head and neck. *Int. J. Radiat. Oncol. Biol. Phys.*, **6**, 1645.

PECKHAM, M.J. & STEEL, G.G. (1972). Cell kinetics in reticulum cell sarcoma. *Cancer*, **29**, 1724.

RICCARDI, A., DANOVA, M., WILSON, G.D. & 8 others (1988). Cell kinetics in human malignancies studied with *in vivo* administration of bromodeoxyuridine and flow cytometry. *Cancer Research*, submitted.

SAKUMA, J. (1980). Cell kinetics of human squamous cell carcinomas in the oral cavity. *Bull. Tokyo Med. Dent. Univ.*, **27**, 43.

SAUNDERS, M.I. & DISCHE, S. (1986). Radiotherapy employing three fractions in each day over a continuous period of 12 days. *Br. J. Radiol.*, **59**, 523.

SCHUTTE, B., REYNDERS, M.M.J., VAN ASSCHE, C.L.M.V.J., HUPPERETS, P.S.G.J., BOSMAN, F.J. & BLIJHAM, G.H. (1987). An improved method for the immunocytochemical detection of bromodeoxyuridine labeled nuclei using flow cytometry. *Cytometry*, **8**, 372.

SHIRAKAWA, S., LUCE, J.K., TANNOCK, I. & FREI, E. (1970). Cell proliferation in human melanoma. *J. Clin. Invest.*, **49**, 1188.

STEEL, G.G. (1977). *Growth kinetics of tumours*. Clarendon Press, Oxford.

TERZ, J.J., CURUTCHET, H.P. & LAWRENCE, W. (1971). Analysis of the cell kinetics of human solid tumours. *Cancer*, **28**, 1100.

THAMES, H.D., PETERS, L.J., WITHERS, H.R. & FLETCHER, G.H. (1983). Accelerated fractionation vs. hyperfractionation: Rationales for several treatments per day. *Int. J. Radiat. Oncol. Biol. Phys.*, **9**, 127.

TROTT, K.-R. & KUMMERMEHR, J. (1985). What is known about tumour proliferation rates to choose between accelerated fractionation or hyperfractionation? *Radiother. Oncol.*, **3**, 1.

WAGNER, H.P. & KASER, H. (1972). Cell proliferation in neuroblastoma. *Eur. J. Cancer*, **6**, 369.

WEISS, W. (1971). The mitotic index in bronchogenic carcinoma. *Am. J. Respir. Dis.*, **104**, 536.

WHITE, R.A. & MEISTRICH, M.L. (1986). A comment on "A method to measure the duration of DNA synthesis and the potential doubling time from a single sample". *Cytometry*, **7**, 486.

WILSON, G.D., McNALLY, N.J., DUNPHY, E., KARCHER, H. & PFRAGNER, R. (1985). The labelling index of human and mouse tumours assessed by bromodeoxyuridine staining *in vitro* and *in vivo* and flow cytometry. *Cytometry*, **6**, 641.

WILSON, G.D., McNALLY, N.J., DISCHE, S. & BENNETT, M.H. (1988). Cell proliferation in human tumours measured by *in vivo* labelling with bromodeoxyuridine. *Br. J. Radiol.*, **61**, 419.

WILSON, G.D., SORANSON, J.A. & LEWIS, A.A. (1987). Cell kinetics of mouse kidney using bromodeoxyuridine incorporation and flow cytometry: Preparation and staining. *Cell Tissue Kinet.*, **20**, 125.

YOUNG, R.C. & DeVITA, V.T. (1970). Cell cycle characteristics of human solid tumours *in vivo*. *Cell Tissue Kinet.*, **3**, 285.